# Efficacy of non-surgical interventions for midfoot osteoarthritis: a systematic review

**DOI:** 10.1007/s00296-023-05324-3

**Published:** 2023-04-24

**Authors:** Polly Q. X. Lim, Merridy J. Lithgow, Michelle R. Kaminski, Karl B. Landorf, Hylton B. Menz, Shannon E. Munteanu

**Affiliations:** 1 Discipline of Podiatry, School of Allied Health, Human Services and Sport, La Trobe University, Melbourne, Victoria 3086 Australia; 2 La Trobe Sport and Exercise Medicine Research Centre, School of Allied Health, Human Services and Sport, La Trobe University, Melbourne, Victoria 3086 Australia; 3 Department of Podiatry, Monash Health, Melbourne, Victoria 3168 Australia; 4 School of Primary and Allied Health Care, Monash University, Melbourne, Victoria 3199 Australia

**Keywords:** Foot, Foot joints, Midfoot, Osteoarthritis, Midfoot osteoarthritis

## Abstract

This systematic review aims to investigate the efficacy of non-surgical interventions for midfoot osteoarthritis (OA). Key databases and trial registries were searched from inception to 23 February 2023. All trials investigating non-surgical interventions for midfoot OA were included. Quality assessment was performed using the National Institutes of Health Quality Assessment Tool. Outcomes were pain, function, health-related quality of life, and adverse events. Effects (mean differences, standardised mean differences, risk ratios) were calculated where possible for the short (0 to 12 weeks), medium (> 12 to 52 weeks), and long (> 52 weeks) term. Six trials (231 participants) were included (one feasibility trial and five case series) — all were judged to be of poor methodological quality. Two trials reported arch contouring foot orthoses to exert no-to-large effects on pain in the short and medium term, and small-to-very-large effects on function in the short and medium term. Two trials reported shoe stiffening inserts to exert medium-to-huge effects on pain in the short term, and small effects on function in the short term. Two trials of image-guided intra-articular corticosteroid injections reported favourable effects on pain in the short term, small effects on pain and function in the medium term, and minimal long term effects. Two trials reported minor adverse events, and none reported health-related quality of life outcomes. The current evidence suggests that arch contouring foot orthoses, shoe stiffening inserts and corticosteroid injections may be effective for midfoot OA. Rigorous randomised trials are required to evaluate the efficacy of non-surgical interventions for midfoot OA.

**Supplementary Information:**

The online version contains supplementary material available at 10.1007/s00296-023-05324-3.

## Introduction

Foot osteoarthritis (OA) is a significant cause of foot pain and disability in older adults [1]. Midfoot OA is one of the most common forms of foot OA affecting 1 in 8 adults aged 50 years and over [1], and is associated with increased age, female sex, lower socioeconomic class, obesity, pain in other weight-bearing joints, the presence of non-musculoskeletal comorbidities, and previous foot and ankle injury [2]. Individuals with midfoot OA report impaired physical [2–4] and mental [2] function compared to individuals without midfoot OA, with over 80% reporting the condition to be disabling [2]. The midfoot is a complex region made of multiple articulations, and midfoot OA has been reported to present as three phenotypes based on the radiographic pattern of joint involvement; medial midfoot (talonavicular, navicular-first cuneiform, or cuneiform-first metatarsal joint), the central midfoot (second cuneiform-metatarsal joint), or both the medial and central midfoot joints [5]. Although midfoot OA is typically described as pain occurring at the dorsal aspect of the midfoot with the presence of radiographic changes of midfoot joints [5, 6], there is no consensus regarding its definition [7].

Currently, there are no evidence-based clinical guidelines to inform the management of midfoot OA. However, non-surgical interventions such as anti-inflammatory and analgesic medications, intra-articular corticosteroid injections, physical therapy, foot orthoses (FOs), and footwear modifications are commonly used as a first line approach to manage foot OA [8, 9]. Non-steroidal anti-inflammatory drugs (NSAIDs) are anti-inflammatory and analgesic agents, primarily exerting their effects by inhibiting prostaglandin synthesis via inhibition of cyclooxygenase (COX) enzymes [10]. Intra-articular corticosteroids [11, 12] are potent anti-inflammatory compounds that exert their effects by acting directly on nuclear steroid receptors [13]. Low-dose radiotherapy has been used for pain relief for foot and ankle osteoarthritis [14], however the mechanism of action is unknown [15]. As alterations in foot and lower limb biomechanics are likely to play an important role in the development and progression of midfoot OA [16], interventions such as FOs and footwear modifications that can alter midfoot joint movement and forces during gait [17–19] are speculated to be effective for midfoot OA [19–22]. Where these non-surgical interventions are unable to improve symptoms, surgery can be considered [9].

As no systematic review of interventions for midfoot OA currently exists, the aim of this study was to systematically review and summarise the evidence relating to studies that have evaluated the efficacy of non-surgical interventions for this condition.

## Methods

### Review registration

This review was prospectively registered with PROSPERO (CRD42021273375) and has been reported in accordance with the Preferred Reporting Items for Systematic Reviews and Meta-analyses (PRISMA) guidelines [23].

### Study inclusion

All trial designs that included adults with midfoot OA confirmed by radiology or physician diagnosis who underwent any non-surgical intervention were eligible for the review. As there is currently no consensus definition for midfoot OA [7], we included all trials that described any symptoms and/ or radiographic degenerative joint changes around the midfoot joints (Supplementary file 1). Trials were excluded if they included participants who: (i) were under 18 years of age, (ii) had neuromuscular or inflammatory arthritic conditions, or (iii) had undergone lower limb surgery. Single case reports, expert opinion pieces, protocols, abstracts without full text, or conference proceedings were excluded.

### Search strategy

A comprehensive literature search was conducted without date restriction up to 16 September 2021 and updated on 23 February 2023 in the following electronic databases: Medline, CINAHL, Embase, the Cochrane Library, and the WHO International Clinical Trials Registry. Two strings of search terms were developed including MeSH terms, keywords, and synonyms: (i) “midfoot” AND “osteoarthritis” and (ii) “non-operative intervention” or “treatment” or “therapy”. Truncation, proximity, and Boolean operators were used as appropriate, and limiters were applied for human studies (Supplementary file 1). In addition to the electronic database search, reference lists of included trials were hand-searched, and citation tracking was performed. There were no date or language restrictions applied.

### Study selection and data extraction

Search results were imported into Endnote 20.1 (Clarivate Analytics, New York, USA) and Covidence (Veritas Health Innovation, Melbourne, Australia), and duplicates were removed. Two investigators (PQXL, SEM) independently screened all titles and abstracts. Full-text articles were obtained if the investigators were not able to determine whether to include the record from the title and abstract. Any disagreements were discussed and resolved by a third investigator (HBM).

The following outcome measures to be extracted were pre-specified before reviewing the articles: (i) pain, (ii) function, (iii) health-related quality of life, (iv) number of participants experiencing any adverse event, and (v) number of participant withdrawals due to adverse events. Outcome measures were obtained for the following time-points: short term (0 to 12 weeks), medium term (> 12 to 52 weeks), long term (> 52 weeks). If two follow-up assessments were completed within one of the defined time-points, the results of the latter of the two assessments were selected. For studies that used multiple measures to evaluate the same outcome (e.g., multiple pain measures), a consensus approach (involving PQXL, SEM, KBL, HBM and MRK) was undertaken to select one outcome measure considered to be the most valid representation of the outcome. This was performed without knowledge of the results for these outcomes to minimise bias. Relevant data were then extracted independently by PQXL and SEM and entered into Microsoft® Excel (Microsoft Corporation, Redmond, Washington, USA) using a standardised extraction form. Attempts were made to contact the authors for missing data, and any disagreements were discussed and resolved by consensus with a third investigator (HBM).

### Quality assessment

The methodological quality of included trials was assessed using the National Institutes of Health (NIH) Quality Assessment Tools [24]. Randomised controlled trials were appraised using the ‘Controlled Intervention’ Tool, which consisted of 14 items. Case series trials were assessed using the ‘Before-After (Pre-Post) Studies With No Control’ Tool. For this tool, one item (‘if the intervention was conducted at a group level’) was excluded as it was considered not applicable to the samples in the trials included in our review, resulting in 11 items of this tool being used. Although the NIH tools [24] allow an overall rating (‘poor’, ‘fair’ or ‘good’) to be applied to each trial, there were no specific scoring thresholds provided to determine the overall rating. Therefore, prior to conducting the quality assessment, a consensus approach (involving PQXL, SEM, KBL, MRK and HBM) was undertaken to determine key criteria that needed to be satisfied to be considered a poor, fair, or good quality trial. For the ‘Controlled Intervention’ Tool, the following items needed to be satisfied: all items for a good quality trial; items 2–8 and 11–14 for a fair quality trial; and none of items 2–8 and 11–14 for a poor quality trial. For the ‘Before-After (Pre-Post) Studies With No Control’ Tool, the following items needed to be satisfied: all items for a good quality trial; items 2, 3, 5, 6, 7, 9, 10 for a fair quality trial; and none of items 2, 3, 5, 6, 7, 9, 10 for a poor quality trial. Quality assessments were undertaken independently by PQXL and SEM, and any disagreements were resolved by consensus with two other investigators (KBL and HBM).

### Data synthesis and analysis

Data were entered into the RevMan software program (V5.4.1; Copenhagen: The Nordic Cochrane Centre, The Cochrane Collaboration, 2014) to obtain estimates of treatment effect. For trials that included a control group, treatment effects from between-group analyses were used. For trials that did not have a control group (i.e., case series), treatment effects from within-group analyses were used. For continuous scaled outcome measures, estimates were analysed as mean differences (MDs) with 95% confidence intervals (CIs) and standardised mean differences (SMDs) with 95% CIs. Results are presented such that a positive MD and SMD value would indicate an effect favouring the experimental intervention (for between-group analyses) or an improvement in an outcome following treatment (for case series trials). SMD effect sizes and 95% CIs were calculated to obtain a measure of the magnitude of differences. SMD values were classified as very small (0.01), small (0.2), medium (0.5), large (0.8), very large (1.2), and huge (2.0) [25]. For dichotomous scaled outcome measures, estimates were analysed as risk ratios with 95% CIs (for between-group analyses) or presented using descriptive statistics (proportions for within-group analyses). A quantitative synthesis (meta-analysis) was not performed as the data from included trials were not sufficiently homogenous due to the variability in trial designs, interventions, and the reported outcomes.

## Results

### Search results

**Fig. 1:**
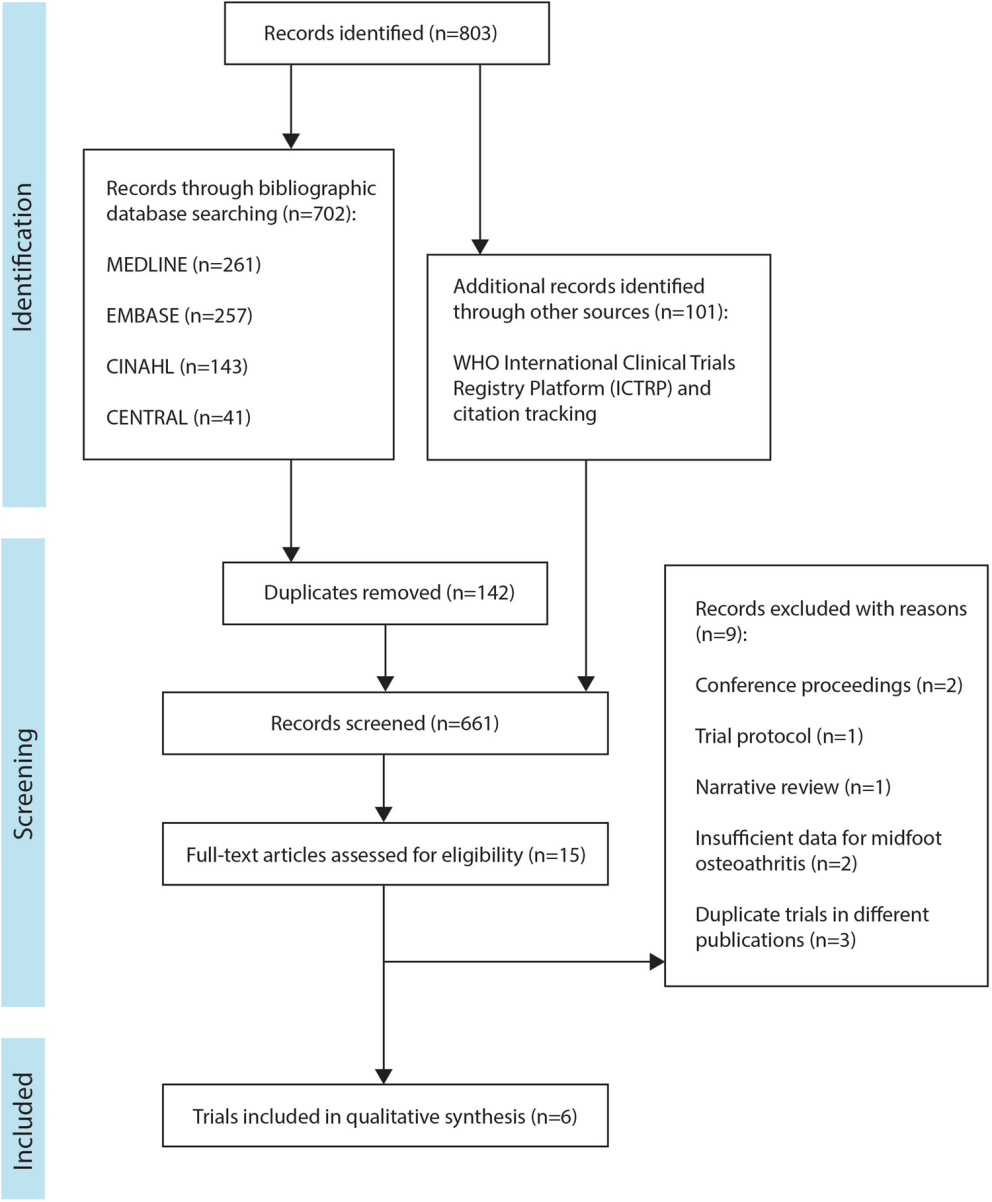
Flowchart of search strategy results

The systematic search identified 803 records. After the removal of duplicates and screening by title and abstract, 15 articles were considered for full-text review. No additional articles were identified in a search through the reference lists and citation tracking of relevant trials. Of the 15 potential articles, nine were excluded (two were conference proceedings, one was a trial protocol, one was a narrative review, two included pooled data for midfoot and ankle OA which could not be separated, and three were trials from the same authors in duplicate publications). A total of six trials met the eligibility criteria and were included in the final review (Fig. 1).

### Characteristics of included trials

The characteristics of these trials are shown in Tables 1 and 2. One trial [20] was a randomised feasibility trial with a control group, and five trials [11, 12, 19, 21, 22] were case series designs. Three trials were from the United Kingdom (UK) [11, 12, 20], one from Australia [22], one from South Korea [19] and one from the United States of America (USA) [21]. A total of 231 participants were included. Sample sizes ranged from 6 to 63 in the case series trials and 37 in the feasibility trial. Overall, participants were typically middle-aged with reported mean age ranges from 53 to 69 years, and more than half (58%) were female (134 women, 97 men). Participants were overweight, with the mean body mass index (BMI) ranging from 29.6 to 31.2 kg/m^2^ in the three trials where it was reported [12, 20, 21].

**Table 1:** Characteristics of included trials with a control group*

Author, year [ref]	Trial design	Country, Population	Groups	Sample size	Age, years (mean ± SD)	Sex, female, n (%)	BMI, kg/m^2^ (mean ± SD)	Unilateral/ bilateral presentation (n/%)	Outcomes measures reported in both groups	Timepoints
Halstead et al. 2016 [20]	Two-arm parallel group, randomised controlled feasibility trial	UK, adults aged ≥ 18 years with radiographically confirmed midfoot OA and symptoms of the dorsal and medial regions of the midfoot	Intervention group (prefabricated arch contouring FOs)	19	60.5 ± 10.4	15 (78.9)	31.2 ± 4.5	Right foot (11/57.9)	Pain measured using NRS (0 to 10) while walking in the last week Function measured using MFPDI function subscale	Baseline, 12 weeks (short term)
			Control group (sham FOs)	18	56.2 ± 12.6	11 (61.1)	27.7 ± 3.9	Right foot (9/50.0)		

*SD standard deviation, *BMI *body mass index, *FOs *foot orthoses, *NRS *numerical rating scale, *MFPDI *Manchester foot pain and disability index

**Table 2:** Characteristics of included trials without a control group*

Author, year [ref]	Trial design	Country, population	Intervention	Sample size	Age, years (mean ± SD)	Sex, female, *n* (%)	BMI, kg/m^2^ (mean ± SD)	Unilateral/ bilateral presentation (*n*/%)	Outcome measures reported	Timepoints
Ibuki et al. 2010 [22]	Case series, prospective	Australia, adults with midfoot OA confirmed with radiographic evidence specifically abnormal MRI, bone scans or x-rays and the presence of pain in the midfoot region	Custom-made arch contouring FOs^†^	57	63.9	41 (71.9)	Not reported	Right foot (14/24.6) Left foot (28/49.1) Bilateral (15/26.3)	Pain measured using NRS (0 to 10) for worst level of pain	Baseline, 3 months (short term), 6 months (medium term)
Rao et al. 2009 [21]	Case series, prospective	USA, adults aged ≥ 18 years with radiographic evidence of degenerative changes at 1 or more tarsometatarsal joints, presence of pain on the dorsum of the foot localised to the tarsometatarsal region aggravated by weight bearing	Shoe stiffening inserts	20	63.0 ± 6.0	20 (100.0)	29.7 ± 5.1	Not reported	Pain measured using FFI-R pain subscale Function measured using FFI-R disability subscale	Baseline, 4 weeks (short term)
Yi et al. 2018 [19]	South Korea, Case series, within session/immediate effects	South Korea, adults with pain on dorsum of the foot (from transverse tarsal joints – talonavicular and calcaneocuboid joints—to the tarsometatarsal region) with exacerbation of pain with weight bearing, and degenerative changes at 1 or more transverse tarsal or tarsometatarsal joints on plain radiographs and/or an increased uptake in the midfoot region on bone scan	Shoe stiffening inserts	7 (mild midfoot arthritis^‡^)	53.3 ± 7.3	5 (71.4)	Not reported^§^	Not reported	Pain measured using VAS (0 to 100) for severity of pain while walking	Baseline, within same session post-treatment
				6 (moderate midfoot arthritis^‡^)	55.5 ± 12.1	4 (66.7)	Not reported^§^	Not reported	Pain measured using VAS (0 to 100) for severity of pain while walking	Baseline, within same session post-treatment
Drakonaki et al. 2011 [11]	Case series, retrospective	UK, adults referred for ultrasound examinations and ultrasound guided intra-articular corticosteroid/local anaesthetic injection in midfoot joints and evidence of degenerative changes from ultrasound and x-ray	Intra-articular corticosteroid injection^¶^	51^#^	65.0 ± 10.6	41 (65.1)	Not provided	Not reported	Pain measured as positive (*better or no pain*) or negative effect (*worse or same*)	No baseline, 1 to 3 months (short term), 6 to 12 months (medium term)
Protheroe et al. 2018 [12]	Case series, retrospective	UK, adults aged ≥ 18 years who underwent x-ray guided intra-articular corticosteroid injections following unsuccessful conservative treatment for physician diagnosed midfoot arthritis	Intra-articular corticosteroid injection^**^	37^††^	68.7 ± 10.1	23 (62.2)	29.6 ± 9.9	Not reported	Function measured using SEFAS (0 to 48)	Baseline, 12 months (medium term)

*SD = standard deviation; BMI = body mass index; FOs = foot orthoses; NRS = numerical rating scale; FFI-R = Foot Function Index-Revised; VAS = visual analogue scale; SEFAS = Self-reported Foot and Ankle Score

^†^Additional carbon fibre footplates were incorporated into the soles of shoes if participants were agreeable (*n* = 36). Participants who did not agree to this shoe modification were advised to wear supportive firm-soled shoes

^‡^The authors divided participants into two subgroups according to the severity of their initial VAS score (0 to 10 scale). Participants in the mild midfoot arthritis group had < 4 VAS pain scores at rest and in the moderate midfoot arthritis group had ≥ 4 VAS scores at rest

^§^The authors reported mean height (161.4 ± 8.4 cm) and weight (63.8 ± 12.8 kg) in the group with mild midfoot arthritis, and mean height (165.2 ± 7.7 cm) and weight (63.8 ± 8.6 kg) in the group with moderate midfoot arthritis

^¶^The authors did not specify if one or multiple intra-articular injections were given

^#^The authors reported that 63 participants were eligible, however data were only available for 51 participants

**The authors reported that some participants received multiple injections determined at the time of the procedure by the consultant orthopaedic foot and ankle surgeon

^††^The authors only reported patient characteristics for 37 out of 41 participants

Two trials evaluated the efficacy of arch contouring FOs [20, 22], two trials evaluated shoe stiffening inserts [19, 21], and two trials evaluated intra-articular corticosteroid injections [11, 12]. The duration of follow-up varied. One trial reported immediate effects [19], four trials [11, 20–22] reported short term outcomes (≤ 12 weeks), three trials [11, 12, 22] reported medium term outcomes (> 12 to 52 weeks), and one trial [11] reported long term outcomes (> 52 weeks).

### Quality assessment

For the one included trial with a control group [20], the quality assessment score was 9 (out of a possible 14), and the trial received an overall rating of ‘poor’ (Supplementary file 2). This trial did not satisfy two criteria: (i) provider blinding was not satisfied as the care provider could not be blinded due to the nature of the intervention (i.e., FOs had to be fitted individually by a clinician), and (ii) the requirement of groups to have similar participant characteristics at baseline was not satisfied as there were differences in BMI and sex at baseline between the intervention and control groups.

For the case series trials [11, 12, 19, 21, 22], the quality assessment score ranged from 2 to 5 (out of a possible 11), and all five trials received an overall rating of ‘poor’ (Supplementary file 2). The most common methodological limitations in these trials related to insufficient sample size (5/5 [100%] of trials), participants not representative of population of interest (5/5 [100%] trials), lack of prespecified inclusion and exclusion criteria (5/5 [100%] trials), lack of assessor blinding (5/5 [100%] trials), insufficient information regarding concomitant treatment (4/5 [80%] trials), lack of information whether interventions and outcome measures were applied consistently throughout (5/5 [100%] trials) and more than 20% loss to follow up (3/5 [60%] trials).

### Effects of interventions

#### Prefabricated arch contouring FOs

One randomised feasibility trial [20] compared prefabricated arch contouring FOs to sham FOs up to 12 weeks (*n* = 37). The arch contouring FOs investigated were VectOrthotic^®^ devices, that could be customised with optional medial rearfoot wedges of 2, 4 or 6 degrees and a VectOrthotic^®^ top cover of 4 to 14 mm. The sham device was a VectOrthotic® 4 mm top cover. Both interventions were provided as a pair and prescribed by an experienced clinician (podiatrist).

Outcomes for pain, function, and adverse events at 12 weeks (short term) were reported. For pain, there were no statistically significant differences between groups. Mean pain (measured using a numerical rating scale [NRS]; scores range from 0 to 10, lower scores indicate less pain whilst walking in the last week) was 4.3 ± 2.1 in the prefabricated arch contouring FOs group and 4.1 ± 2.1 in the sham FOs group at 12 weeks (MD  – 0.20, 95% CI  – 1.57 to 1.17, *p* = 0.78, SMD = – 0.09). For function, there were no statistically significant differences between groups. Mean function (measured using the Manchester Foot Pain and Disability Index [MFPFI]; scores range from 0 to 34, lower scores indicate better foot function) was 6.5 ± 4.7 in the prefabricated arch contouring FOs group and 8.4 ± 5.2 in the sham FOs group at 12 weeks (MD 1.90, 95% CI  – 1.34 to 5.14, *p* = 0.25, SMD = 0.37) (Table 3).

**Table 3:** Between-group data for pain, function, and adverse events (if any) in the short, medium, and long term*

Author, year [ref]	Intervention	Outcome	Timepoint reported	Outcome measure	Experimental group Mean ± SD (*n*)	Comparator group Mean ± SD (*n*)	Mean difference^†^ (95% CI), *p*-value	Standardised mean difference^†^ (95% CI)
Halstead et al. 2016 [20]	Prefabricated arch contouring FOs versus sham FOs	Pain	Short term, 12 weeks	NRS while walking in last week	4.3 ± 2.1 (18)	4.1 ± 2.1 (18)	– 0.20 ( – 1.57, 1.17), 0.78	-0.09 (-0.75, 0.56)
		Function	Short term, 12 weeks	MFPDI^‡^	6.5 ± 4.7 (18)	8.4 ± 5.2 (18)	1.90 ( – 1.34, 5.14), 0.25	0.37 ( – 0.28, 1.03)
		Adverse events	Short term, 12 weeks	Number of reported adverse events	Number of adverse events / total in experiment group (%) 1/19 (5.3)	Number of adverse events / total in comparator group (%) 2/18 (11.1)	Risk ratio (95% CI), *p*-value 0.47 (0.05, 4.78), 0.53	

*SD standard deviation, *CI *confidence intervals, *FOs *foot orthoses, *NRS *numerical rating scale, *MFPDI* Manchester foot pain and disability index

^†^Positive mean difference and standardised mean difference values favour the experimental group

^‡^Lower MFPDI scores represent better function

No adverse events in the participants who completed the trial were reported. One participant from the arch contouring FOs group (1/19 [5.3%]) withdrew due to escalating back pain and burning pain in their feet. Two participants from the sham FOs group (2/18 [11.1%]) were unable to complete the trial due to unrelated back pain. The difference in risk of participants withdrawing from adverse events between the two groups was not statistically significant (RR 0.47, 95% CI 0.05 to 4.78, *p* = 0.53) (Table 3). No outcomes relating to health-related quality of life or serious adverse events were reported.

#### Custom-made arch contouring FOs

One case series trial [22] investigated the efficacy of custom-made arch contouring FOs up to 6 months (*n* = 57). The FOs were constructed from full-length ethylene vinyl acetate (EVA) of two densities (220 kg/m^3^ first layer and 270 kg/m^3^ second layer). Participants were offered rigid carbon fibre footplates that were incorporated into the soles of their shoes in addition to the FOs, and 36 participants received this modification.

Outcomes for pain and function at 3 months (short term) and 6 months (medium term) were reported. For pain, there was a statistically significant improvement from baseline at 3 and 6 months. Mean pain (measured using an NRS; scores range from 0 to 10 scale, lower scores indicate less pain) was 7.81 ± 1.65 at baseline, 5.55 ± 2.15 at 3 months (MD 2.26, 95% CI 1.56 to 2.96, *p* < 0.01, SMD = 1.17) and 4.54 ± 2.33 at 6 months (MD 3.27, 95% CI 2.53 to 4.01, *p* < 0.01, SMD = 1.61). For function, there was a statistically significant improvement in function (difficulty in walking outdoors) from baseline at 3 and 6 months. Mean function (measured using an NRS; scores range from 0 to 10, lower scores indicate better function) was 7.81 ± 2.69 at baseline, 4.12 ± 2.54 at 3 months (MD 3.69, 95% CI 2.73 to 4.65, *p* < 0.01, SMD = 1.40) and 4.07 ± 2.73 at 6 months (MD 3.74, 95% CI 2.75 to 4.73, *p* < 0.01, SMD = 1.37) (Table 4). No outcomes relating to health-related quality of life, or adverse events were reported.

**Table 4:** Within-group data for pain, function, and adverse events (if any) in the short, medium, and long term*

Author, year [ref]	Intervention	Outcome	Timepoint reported	Outcome measure	Mean ± SD (*n*) at baseline	Mean ± SD (*n*) at follow-up	Mean difference^†^ (95% CI), *p*-value	Standardised mean difference^†^ (95% CI)
Ibuki et al. 2010 [22]	Custom-made arch contouring FOs^‡^	Pain	Short term, 3 months	NRS worst level of pain	7.81 ± 1.65 (57)	5.55 ± 2.15 (57)	2.26 (1.56, 2.96), < 0.01	1.17 (0.77, 1.57)
			Medium term, 6 months	NRS worst level of pain	7.81 ± 1.65 (57)	4.54 ± 2.33 (57)	3.27 (2.53, 4.01), < 0.01	1.61 (1.18, 2.03)
		Function	Short term, 3 months	NRS difficulty walking outdoors	7.81 ± 2.69 (57)	4.12 ± 2.54 (57)	3.69 (2.73, 4.65), < 0.01	1.40 (0.99, 1.81)
			Medium term, 6 months	NRS difficulty walking outdoors	7.81 ± 2.69 (57)	4.07 ± 2.73 (57)	3.74 (2.75, 4.73), < 0.01	1.37 (0.96, 1.78)
Rao et al. 2009 [21]	Shoe stiffening inserts	Pain	Short term, 4 weeks	FFI-R pain subscale^§^	37.1 ± 9.5 (20)	30.6 ± 10.1 (20)	6.50 (0.42,12.58), 0.04	0.65 (0.01, 1.29)
		Function	Short term, 4 weeks	FFI-R disability subscale^¶^	37.3 ± 10.7 (20)	35.0 ± 11.6 (20)	2.30 ( – 4.62, 9.22), 0.51	0.20 ( – 0.42, 0.82)
Yi et al. 2018 [19]	Shoe stiffening inserts	Pain	Immediate^#^, Within same session	VAS while walking (with mild midfoot arthritis)	2.0 ± 1.0 (7)^**^	2.0 ± 1.0 (7)^**^	0.00 ( – 1.05, 1.05), 1.00	0.00 ( – 1.05, 1.05)
				VAS while walking (with moderate midfoot arthritis)	5.5 ± 1.4 (6)^**^	2.0 ± 0.5 (6)^**^	3.50 (2.31, 4.69), < 0.01	3.07 (1.19, 4.95)
Drakonaki et al. 2011 [11]	Intra-articular corticosteroid injection	Pain	Short term, Between 1 to 3 months	Improvement in pain was measured as a positive or negative effect^††^		23/40 (57.5%) reported a positive effect^††^		
			Medium term, Between 6 to 12 months			4/40 (10.0%) reported a positive effect^††^		
			Long term, > 12 months			1/40 (2.5%) reported a positive effect^††^		
Protheroe et al. 2018 [12]	Intra-articular corticosteroid injection	Pain and function	Medium term, 12 months	SEFAS^‡‡^	17 ± 6.98 (37)	21.3 ± 10.91 (37)	4.30 (0.13, 8.47), 0.04	0.46 (0.00, 0.93)
		Adverse events	Immediate, Up to 3 days	Number of reported adverse events		5/37 (13.5%) participants reported a flare reaction^§§^ 3/37 (8.1%) participants reported post-injection swelling, all resolving after 2 to 3 days^§§^		

*SD standard deviation, *CI *confidence intervals, *FFI* Foot Function Index-Revised, *NRS *numerical rating scale, *VAS *visual analogue scale; *SEFAS *Self-reported Foot and Ankle Score

^†^Positive mean difference and positive standard mean difference values indicate an improvement in that outcome

^‡^Included participants who had their shoes modified with embedded carbon plates in addition to the FOs

^§^Lower scores in the FFI-R pain subscale represent improved pain

^¶^Lower scores in the FFI-R disability subscale represent better function

^#^Immediate time point refers to effects reported within a same-day session

**Baseline VAS was the VAS reported walking in athletic shoes without an insert, follow-up VAS was the VAS reported walking with a shoe stiffening insert

^††^Positive effect was graded as ‘better or no pain’ indicating partial or complete pain relief; negative effect was graded as ‘same or worse’ indicating no pain relief or increase in pain. Mean (SD) values were not reported as the authors reported dichotomous values at follow up

^‡‡^Higher SEFAS scores represent better function

^§§^Mean (SD) values were not reported as the authors reported dichotomous values at follow up

#### Shoe stiffening inserts

One case series trial [19] investigated the immediate effects of shoe stiffening inserts. They were 1.6 mm thick, full-length carbon fibre (FCF) inserts (Schein Orthopädic Service, Remscheid, Germany) with a 2 mm Plastazote® top cover, and a built-in height of 1 cm and toe spring of 1 cm. The participants were separated into ‘mild’ midfoot arthritis (pain severity using a visual analogue scale [VAS] of 1 to 3 out of 10, *n* = 7) and ‘moderate’ midfoot arthritis (pain severity using VAS of 4 to 7 out of 10, *n* = 6) groups. Each group was provided athletic footwear to wear while walking on a 5 m walkway five times with and without the FCF insert. Pain outcomes within a single session (immediate) timepoint were reported. In the group with mild midfoot OA, there were no statistically significant differences in pain levels while walking with and without the FCF insert. Mean pain (measured using a VAS 0 to 10 scale; lower scores indicate less pain) was 2.0 ± 1.0 with the FCF insert and 2.0 ± 1.0 without the FCF insert (MD 0.00, 95% CI  – 1.05 to 1.05, *p* = 1.00, SMD = 0.00). In the group with moderate midfoot OA, there was a statistically significant difference with and without the FCF insert, favouring the intervention. Mean pain was 5.5 ± 1.4 with the FCF insert and 2.0 ± 0.5 without the FCF insert (MD 3.50, 95% CI 2.31 to 4.69, *p* < 0.01, SMD = 3.07) (Table 4). No outcomes pertaining to health-related quality of life, or adverse events were reported.

Another case series trial [21] investigated the effects of shoe stiffening inserts up to 4 weeks (*n* = 20). The inserts were full-length semi-rigid 1.6 mm thick carbon graphite without an arch build up or a heel cup. Pain and function outcomes at 4 weeks (short term) were reported. For pain, there was a statistically significant improvement from baseline at 4 weeks. Mean pain (measured using the pain subscale of the Foot Function Index-Revised [FFI-R] on a 0 to 100 scale; lower scores indicate less pain) was 37.1 ± 9.5 at baseline and 30.6 ± 10.1 at 4 weeks (MD 6.50, 95% CI 0.42 to 12.58, *p* = 0.04, SMD = 0.65). For function, there was no statistically significant difference from baseline to 4 weeks. Mean function (measured using the disability subscale of the FFI-R on a 0 to 100 scale; lower scores indicate better function) was 37.3 ± 10.7 at baseline and 35.0 ± 11.6 at 4 weeks (MD 2.30, 95% CI  – 4.62 to 9.22, *p* = 0.51, SMD = 0.20) (Table 4). No outcomes relating to health-related quality of life, or adverse events were reported.

#### Intra-articular corticosteroid injection

One case series trial [11] investigated effects of ultrasound guided intra-articular corticosteroid injections for more than a year (*n* = 63). A long-acting anaesthetic solution of 1 to 1.5ml (bupivacaine hydrochloride 0.5%, Marcain Polyamp Steripack 0.5%, 10 ml, AstraZeneca, Luton, UK) combined with 20 to 40 mg of corticosteroid (methylprednisolone acetate BP, Depomedrone 1 ml, 40 mg/vial, Pharmacia Ltd, Kent, UK) was injected into the affected joint in the midfoot region. Participants were provided pain diaries to complete over 2 weeks. Pain outcomes for between 1 to 3 months (short term), between 3 to 12 months (medium term) and after 12 months (long term) timepoints were reported. Pain was evaluated by reviewing the clinical notes post-injection as a positive effect (*better or no pain* indicating complete or partial pain relief) and a negative effect (*same or worse* indicating no pain relief or an increase in the amount of pain). For pain, 23 out of 40 (57.5%) participants had an improvement in pain between 1 to 3 months, 4 (10%) participants had an improvement in pain between 6 to 12 months, and 1 (2.5%) participant had an improvement in pain after 12 months (see Table 4). No outcomes pertaining to function, health-related quality of life, or adverse events were reported.

Another case series trial [12] investigated the effects of x-ray guided intra-articular injections up to 12 months (*n* = 41). Participants received an injection of 1 mL of 2% lidocaine followed by 80 mg methylprednisolone (Depomedrone) and 2 mL 0.5% levobupivacaine per area under x-ray guidance (Vertec, C arm). The number of joint injections given was determined by the orthopaedic foot and ankle surgeon administering the injections, but the number of injections provided to each participant was not reported. Outcomes for pain and function combined at 12 months (medium term) were reported using the Self-reported Foot and Ankle Score (SEFAS) questionnaire. There was a statistically significant improvement in pain and function at 12 months. Mean pain and function (measured using the SEFAS; scores range from 0 to 48, higher scores indicate less pain and better function) was 17 ± 6.98 at baseline and 21.3 ± 10.91 at 12 months (MD 4.30, 95% CI 0.13 to 8.47, *p* = 0.04, SMD = 0.46) (see Table 4). Minor adverse events were reported. Five (14%) participants reported a flare reaction, and three (8%) participants reported post-injection swelling, all resolving after 2 to 3 days. No withdrawal of participants due to adverse events was reported, and the authors did not report any outcomes pertaining to function or health-related quality of life.

## Discussion

This systematic review aimed to summarise the evidence for the efficacy of non-surgical interventions for midfoot OA. There are no randomised controlled trials – the existing trials are limited to a feasibility trial or case series trials. However, the current, albeit limited, evidence indicates that arch contouring FOs, shoe stiffening inserts and corticosteroid injections may be effective for improving pain and/or function in midfoot OA. The effects of interventions for midfoot OA on health-related quality of life are unknown. Further, there is limited evidence regarding the harms of interventions for midfoot OA, as few trials reported adverse events.

Two trials investigated the efficacy of arch-contouring FOs [20, 22]. Arch contouring FOs are used to support the midfoot with a close-fitting orthotic shell and increase contact time and maximum force underneath the midfoot [18, 20]. This is theorised to reduce bending moments across the midfoot joints, prevent medial longitudinal arch deformation and reduce midfoot joint compression and pain [18, 26]. Arch contouring FOs were found to have none [20] to large [22] effects on pain in the short term (≤ 12 weeks), very large [22] effects on pain in the medium term (> 12 to 52 weeks), medium [20] to very large [22] effects on function in the short term (≤ 12 weeks), and very large [22] effects on function in the medium term (> 12 to 52 weeks). Although it appears that arch contouring FOs have very large effects in one trial [22], the authors reported that a proportion of participants (36 out of 57) had additional rigid carbon fibre plates incorporated into the soles of their shoes. This could have potentiated the effects of the intervention. Overall, arch contouring FOs are well tolerated, with only one participant reporting an adverse event in one trial [20]. However, the methodological quality of these trials was judged to be poor and the effects beyond 52 weeks have not been investigated. Appropriately powered randomised trials are required to determine the effects of arch contouring foot orthoses for midfoot OA.

The efficacy of shoe stiffening inserts was investigated in two trials [17, 19]. Shoe stiffening inserts have been found to reduce first metatarsal joint dorsiflexion and first metatarsal plantarflexion during gait [17]. As the proximal aspects of the first, second and third metatarsals form part of the distal articulation of the tarsometatarsal joint, reducing movement of the metatarsals is theorised to limit articular stress within the midfoot and potentially reduce midfoot joint pain [17]. Shoe stiffening inserts were found to have no effects [19] in reducing pain in individuals with mild midfoot OA in the immediate timepoint (within a session), huge effects [19] in reducing pain in individuals with moderate midfoot OA in the immediate timepoint, medium effects [21] on pain improvement in the short term (≤ 12 weeks), and small effects [21] on function in the short term (≤ 12 weeks). Shoe stiffening inserts are well tolerated with one trial [21] reporting no adverse events. However, these trials were judged to be of poor methodological quality and the effects of the intervention beyond 12 weeks are unknown.

The efficacy of image-guided intra-articular corticosteroid injection was investigated in two trials [11, 12]. Corticosteroid injections are common anti-inflammatory pharmacological agents used for inflammation and pain management in ankle and knee osteoarthritis [27, 28]. In the included trials, methylprednisolone was the primary agent used and was injected into midfoot joints guided by ultrasound [11] or x-ray [12]. Corticosteroid injection was found to provide short term (≤ 12 weeks) improvement in pain and function with heterogenous effects that ranged from small to large [11, 12]. These intervention effects were not maintained in the medium (> 12 to 52 weeks) and longer term (> 52 weeks). The observed pattern of efficacy in the short term, but reducing in the medium and long term, is consistent with findings reported in trials evaluating corticosteroid injection for other musculoskeletal conditions of the foot such as plantar heel pain and tendon disorders [29–31]. This suggests that corticosteroid injection alone may have limited utility for midfoot OA. Adverse events associated with intra-articular corticosteroid injections were reported in both trials and were uncommon (0 to 13.5%) and minor [11, 12]. As the included trials were rated as poor quality, rigorous randomised trials are required to improve our understanding of the efficacy of corticosteroid for midfoot OA.

There are several strengths of this review. The review protocol was prospectively registered with PROSPERO. We used a robust search strategy that was comprehensive and not restricted by language or date. We used two independent investigators to screen studies for inclusion, perform data extraction and conduct quality assessment with conflicts resolved by two investigators. The reporting of data and results were cross checked. We also used pre-determined decision rules to identify and extract data for outcome measures at specific time points. Furthermore, we pre-specified important criteria within the NIH Quality Assessment Tools [24] that had to be satisfied prior to rating the quality for each trial. As such, our assessments regarding the methodological quality of the evidence are transparent and unbiased.

The quality of the evidence in this review is limited by the small number and low level of evidence of the included trials, and their poor methodological quality. Our quality assessment identified several common issues across the included trials, so all were rated as poor quality. First, the inclusion criteria for the diagnosis of midfoot OA were unclear in most trials [11, 12, 19, 21, 22] which makes it difficult to generalise the findings. Further work is required to develop a consensus definition for midfoot OA [7]. Second, the outcome measures varied in type and assessment timepoints across the trials, and only three trials [12, 20, 21] used validated measures for pain and function [32]. Third, there was a lack of participant and assessor blinding across all trials [11, 12, 19, 21, 22] apart from the feasibility trial [20]. There was considerable intervention variability in the provision and types of FOs used, and the intra-articular corticosteroid injections performed were guided by different imaging techniques and operators. As such, comparisons could not be made between the included trials. Finally, only the feasibility trial [20] provided a prospective sample size calculation, so it is possible that the remaining trials [11, 12, 19, 21, 22] may have been under-powered leading to an uncertainty in the observed intervention effect [33]. Although the authors [20] reported appropriate sample size calculations adequate for a feasibility trial, key characteristics of participants in each group at baseline were notably different, and this may have confounded the reported effect of the intervention.

In conclusion, the aim of this review was to assess the current evidence for non-surgical interventions for midfoot OA. The available evidence indicates that arch contouring FOs may reduce pain and improve function in the short and medium term, and shoe stiffening inserts may improve pain and function in the short term. However, the long-term effects of these interventions are unknown. Although intra-articular corticosteroid injection therapy may reduce pain in the short term, the effects do not persist. Overall, the quality of evidence that these conclusions are drawn from is low. Therefore, rigorous randomised trials are required to evaluate the efficacy of these non-surgical interventions.

## Supplementary Information

Below is the link to the electronic supplementary material.

Supplementary file1 (DOCX 60 KB)

Supplementary file2 (DOCX 63 KB)

## Data Availability

The data presented in this review are included in the article and supplementary data files. Any additional data will be shared on reasonable request to the corresponding author (PQXL).
