# Supplementary material for: Efficacy of non-surgical interventions for midfoot osteoarthritis: a systematic review
Source: Rheumatol Int. 2023 Apr 24;43(8):1409–22. doi: 10.1007/s00296-023-05324-3 (PMC10261166; doi:10.1007/s00296-023-05324-3)
Supplement: Supplementary file 1 — Supplementary file1 (DOCX 60 KB) [file 296_2023_5324_MOESM1_ESM.docx]

**Supplementary file 1.** Initial search strategy.

Electronic bibliographic databases searched from inception: MEDLINE, EMBASE, CINAHL, Cochrane Central Register of Controlled Trials (CENTRAL), and the World Health Organisation International Clinical Trials Registry Platform (ICTRP).

**MEDLINE (OVID) 1946 – present**

1. Midfoot.mp.

2. Tarsal.mp.

3. Midtarsal.mp.

4. Tarsometatarsal.mp.

5. Tarso-metatarsal.mp.

6. exp Tarsal Joints/

7. 1 or 2 or 3 or 4 or 5 or 6

8. Osteoarthr*.mp.

9. Degenerative joint disease*.mp.

10. (degenerative adj2 arthritis).mp.

11. exp Osteoarthritis/

12. Arthritis/

13. 8 or 9 or 10 or 11 or 12

14. ((nonoperative or non-operative or nonsurgical or non-surgical) adj2 (therap* or treatment*)).mp.

15. (nonoperative or non-operative or nonsurgical or non-surgical) adj2 intervention*.mp.

16. Physical therap*.mp.

17. exp Therapeutics/

18. exp "Physical and Rehabilitation Medicine"/

19. Taping.mp.

20. Foot ortho*.mp.

21. Shoe insole*.mp.

22. Shoe insert*.mp.

23. Footplate*.mp.

24. Foot plate*.mp.

25. exp Orthotic Devices/

26. Shoe*.mp.

27. Footwear.mp.

28. exp Shoes/

29. Corticosteroid*.mp.

30. Injection*.mp.

31. 14 or 15 or 16 or 17 or 18 or 19 or 20 or 21 or 22 or 23 or 24 or 25 or 26 or 27 or 28 or 29 or 30

32. 7 and 13 and 31

33. limit 32 to humans

**EMBASE (OVID) 1947 – present**

1. Midfoot.mp.

2. Tarsal.mp.

3. Midtarsal.mp.

4. Tarsometatarsal.mp.

5. Tarso-metatarsal.mp.

6. exp tarsal joint/

7. exp tarsometatarsal joint/

8. 1 or 2 or 3 or 4 or 5 or 6 or 7

9. Osteoarthr*.mp.

10. Degenerative joint disease*.mp.

11. (degenerative adj2 arthritis).mp.

12. exp osteoarthritis/

13. arthritis/

14. 9 or 10 or 11 or 12 or 13

15. ((nonoperative or non-operative or nonsurgical or non-surgical) adj2 (therap* or treatment*)).mp.

16. (nonoperative or non-operative or nonsurgical or non-surgical) adj2 intervention*.mp.

17. Physical therap*.mp.

18. exp physiotherapy/

19. exp physical medicine/

20. Taping.mp.

21. exp athletic tape/

22. Foot ortho*.mp.

23. Shoe insole*.mp.

24. Shoe insert*.mp.

25. Footplate*.mp.

26. Foot plate*.mp.

27. exp orthosis/

28. exp orthotics/

29. Shoe*.mp.

30. Footwear.mp.

31. exp shoe/

32. Corticosteroid*.mp.

33. exp corticosteroid/

34. Injection*.mp.

35. 15 or 16 or 17 or 18 or 19 or 20 or 21 or 22 or 23 or 24 or 25 or 26 or 27 or 28 or 29 or 30 or 31 or 32 or 33 or 34

36. 8 and 14 and 35

37. limit 36 to human

**CINAHL (EBSCOhost) 1982 – present**

S1. Midfoot

S2. Tarsal

S3. Midtarsal

S4. Tarsometatarsal

S5. “Tarso-metatarsal”

S6. (MH "Tarsal Joint+")

S7. S1 OR S2 OR S3 OR S4 OR S5 OR S6

S8. Osteoarthr*

S9. Degenerative joint disease*

S10. degenerative N2 arthritis

S11. (MH "Osteoarthritis+")

S12. (MH "Arthritis")

S13. S8 OR S9 OR S10 OR S11 OR S12

S14. Physical therap*

S15. (MH “Therapeutics+”)

S16. Taping

S17. Foot ortho*

S18. Shoe insole*

S19. Shoe insert*

S20. Footplate*

S21. Foot plate*

S22. (MH “Orthoses+”)

S23. Shoe*

S24. Footwear

S25. (MH "Shoes+")

S26. (MH "Orthopedic Footwear")

S27. Injection*

S28. (MH "Injections, Intraarticular")

S29. Corticosteroid*

S30. S14 OR S15 OR S16 OR S17 OR S18 OR S19 OR S20 OR S21 OR S22 OR S23 OR S24 OR S25 OR S26 OR S27 OR S28 OR S29

S31. S7 AND S13 AND S30

**Cochrane Central Register of Controlled Trials (CENTRAL)**

#1. Midfoot

#2. Tarsal

#3. Midtarsal

#4. Tarsometatarsal

#5. Tarso-metatarsal

#6. MeSH descriptor: [Tarsal Joints] explode all trees

#7. #1 OR #2 OR #3 OR #4 OR #5 OR #6

#8. Osteoarthr*

#9. Degenerative arthr*

#10. Degenerative joint disease*

#11. MeSH descriptor: [Osteoarthritis] explode all trees

#12. #8 OR #9 OR #10 OR #11

#13. #7 AND #12

**World Health Organisation International Clinical Trials Registry Platform (ICTRP)**

1. ‘Foot’ and ‘Osteoarthritis’
