# Supplementary material for: Efficacy of non-surgical interventions for midfoot osteoarthritis: a systematic review
Source: Rheumatol Int. 2023 Apr 24;43(8):1409–22. doi: 10.1007/s00296-023-05324-3 (PMC10261166; doi:10.1007/s00296-023-05324-3)
Supplement: Supplementary file 2 — Supplementary file2 (DOCX 63 KB) [file 296_2023_5324_MOESM2_ESM.docx]

**Supplementary file 2.** Methodological quality assessment of included trials.

Methodological quality assessment for controlled trials using the NIH Quality Assessment of Controlled Intervention Studies.

|  | RCT description | Adequate randomisation | Allocation concealment | Participant and provider blinding | Assessor blinding | Groups similar at baseline | Drop-out rate ≤ 20% | Differential drop-out rate ≤ 15% (between treatment groups) | Intervention adherence | Other interventions avoided or similar in the groups | Valid and reliable outcome measures | Sample size sufficiently large to detect power ≥ 80% | Outcomes reported or subgroups analysed were prespecified | Intention-to-treat analysis | **Total** | **Quality** |
| --- | --- | --- | --- | --- | --- | --- | --- | --- | --- | --- | --- | --- | --- | --- | --- | --- |
| Halstead (2016) | **+** | **+** | **+** | **-** | **+** | **-** | **+** | **+** | **+** | **?** | **+** | **?** | **+** | **N/A*** | **9** | **Poor** |

* Intention-to-treat analysis not applicable to a randomised feasibility trial as the primary focus is on feasibility outcomes and not treatment measures

Methodological quality assessment for case series trials using the NIH Quality Assessment Tool for Before -After (Pre-Post) Studies With No Control Group.

|  | Research question and objective | Study population defined | Participants are representative of population  of interest | Eligible participants met prespecified criteria | Inclusion / exclusion prespecified | Consistent intervention delivered | Consistent outcome measures assessed | Assessor blinding | < 20% loss to follow-up | Statistical methods provided p values for  outcome measure pre-post intervention | Sample size sufficiently large (detect ≥ 80% power) | **Total** | **Quality** |
| --- | --- | --- | --- | --- | --- | --- | --- | --- | --- | --- | --- | --- | --- |
| Rao (2010) | **+** | **+** | **-** | **+** | **?** | **?** | **?** | **-** | **?** | **+** | **-** | **4** | **Poor** |
| Ibuki (2010 | **+** | **+** | **?** | **?** | **?** | **-** | **?** | **-** | **-** | **+** | **-** | **3** | **Poor** |
| Yi (2018) | **+** | **+** | **?** | **?** | **?** | **+** | **?** | **-** | **+** | **+** | **-** | **5** | **Poor** |
| Drakonaki (2011) | **+** | **-** | **?** | **+** | **?** | **?** | **?** | **-** | **-** | **-** | **-** | **2** | **Poor** |
| Protheroe (2018) | **+** | **-** | **?** | **+** | **?** | **?** | **?** | **-** | **+** | **+** | **-** | **4** | **Poor** |
| **Total items satisfied (100%)** | **5 (100%)** | **3 (60%)** | **0 (0%)** | **3 (60%)** | **0 (0%)** | **1  (20%)** | **0 (0%)** | **0 (0%)** | **2 (40%)** | **4 (80%)** | **0 (0%)** |  |  |
